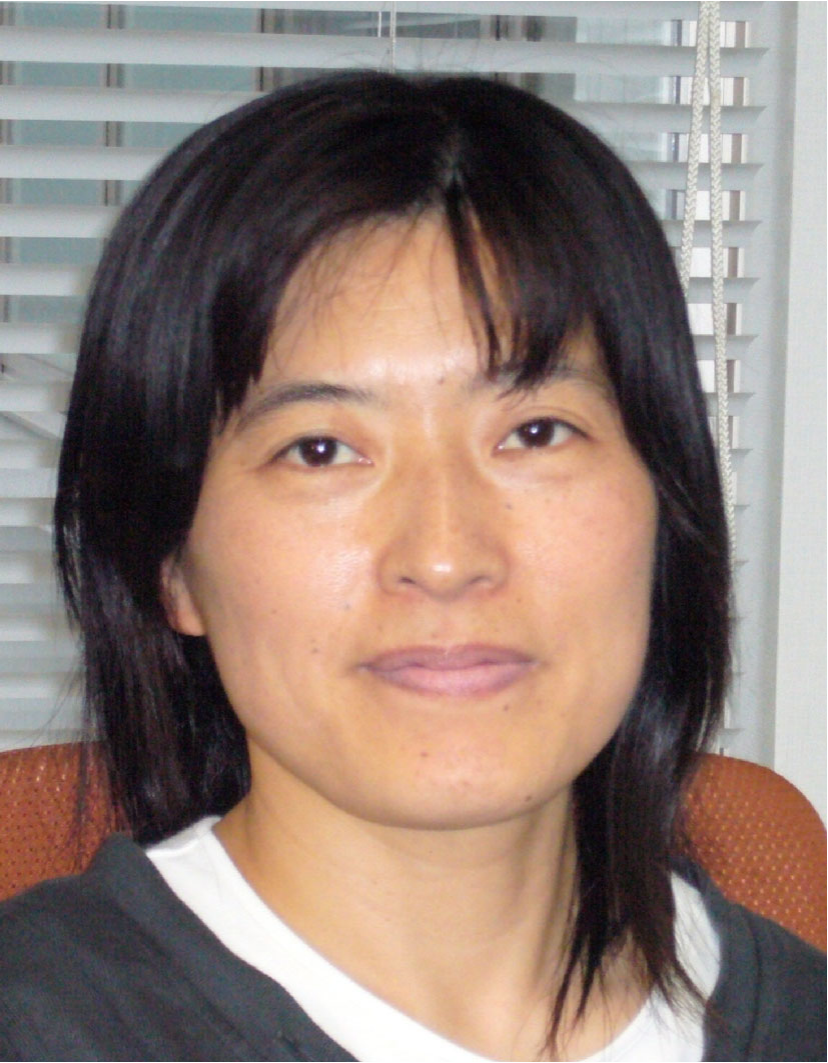# Message From the New Editor-in-Chief

**DOI:** 10.2188/jea.JE20140034

**Published:** 2014-03-05

**Authors:** Manami Inoue

It is a great honor to assume the role of Editor-in-Chief of the Journal of Epidemiology from Dr. Tomotaka Sobue. Under Dr. Sobue’s outstanding leadership, the average impact factor reached 2.113 in 2012, and the expansion of the Journal beyond its original base has continued to accelerate. Indeed, the Journal is now recognized as an international rather than a domestic journal, and manuscripts from abroad account for two-thirds of the total. On behalf of the Board, I wish to thank Dr. Sobue for his inspiring dedication and service.

Like many journals, the Journal of Epidemiology has recently faced major challenges in the global publishing environment. One broad-reaching change has been the introduction of open access licensing. Our adoption of the Creative Commons Attribution License (CC BY v3.0) for all papers allows authors to retain ownership of the copyright of their papers, but also allows anyone to download, reuse, copy, reprint, distribute, or modify the paper, provided they cite the original authors and source. The global change in policy towards open access for papers supported by research grants is unstoppable, and our decision to adopt this model is critical to the future of the journal.

Second, the Editorial Board warmly welcomes the participation of five new associate editors from abroad, starting from 2014. We believe these new colleagues will contribute to the continuing globalization of the Journal.

The editorial team seeks to maintain the Journal’s high international standards and will continue to publish original and review articles on a wide range of topics. Looking to the future, our goal is to establish the Journal as one of the leading journals in epidemiology. I warmly welcome the participation and support of every reader in attaining this goal.

Manami Inoue, MD, PhD Editor-in-Chief Journal of Epidemiology  Project Professor AXA Department of Health and Human Security Graduate School of Medicine The University of Tokyo

**Figure:**